# Acute respiratory distress syndrome subphenotypes and therapy responsive traits among preclinical models: protocol for a systematic review and meta-analysis

**DOI:** 10.1186/s12931-020-01337-9

**Published:** 2020-04-07

**Authors:** Adrien Carla, Bruno Pereira, Hanifa Boukail, Jules Audard, Nathalie Pinol-Domenech, Manuela De Carvalho, Raiko Blondonnet, Ruoyang Zhai, Dominique Morand, Céline Lambert, Vincent Sapin, Lorraine B. Ware, Carolyn S. Calfee, Julie A. Bastarache, John G. Laffey, Nicole P. Juffermans, Lieuwe D. Bos, Antonio Artigas, Patricia R. M. Rocco, Michael A. Matthay, Daniel F. McAuley, Jean-Michel Constantin, Matthieu Jabaudon, for the ESICM Translational Biology Group of the Acute Respiratory Failure section

**Affiliations:** 1 Department of Perioperative Medicine, CHU Clermont-Ferrand, Clermont-Ferrand, France; 2 Biostatistics Unit, Department of Clinical Research and Innovation (DRCI), CHU Clermont-Ferrand, Clermont-Ferrand, France; 3 GReD, CNRS UMR 6293, INSERM U1103, Université Clermont Auvergne, Clermont-Ferrand, France; 4 Université Clermont Auvergne, Health Library, Clermont-Ferrand, France; 5 Department of Medical Biochemistry and Molecular Biology, CHU Clermont-Ferrand, Clermont-Ferrand, France; 6 Division of Allergy, Pulmonary, and Critical Care Medicine, Department of Medicine, Vanderbilt University Medical Center, Nashville, TN USA; 7 Department of Pathology, Microbiology, and Immunology, Vanderbilt University Medical Center, Nashville, TN USA; 8 Division of Pulmonary and Critical Care Medicine, Departments of Medicine and Anesthesia, Cardiovascular Research Institute, University of California San Francisco, San Francisco, CA USA; 9 Department of Cell and Developmental Biology, Vanderbilt University, Nashville, TN USA; 10 Keenan Research Centre for Biomedical Science, Hospital for Sick Children, Departments of Anesthesia and Critical Care Medicine, St. Michael’s Hospital, Departments of Anesthesia, Physiology and Interdepartmental Division of Critical Care, University of Toronto, Toronto, Canada; 11 Regenerative Medicine Institute at CÚRAM Centre for Research in Medical Devices, National University of Ireland Galway, Galway, Ireland; 12 Department of Intensive Care Medicine, Department of Respiratory Medicine, and Laboratory of Experimental Intensive Care and Anesthesiology, Amsterdam University Medical Center, Amsterdam, The Netherlands; 13 Corporació Sanitaria Parc Tauli, CIBER de Enfermedades Respiratorias, Autonomous University of Barcelona, Barcelona, Spain; 14 Laboratory of Pulmonary Investigation, Carlos Chagas Filho Institute of Biophysics, Federal University of Rio de Janeiro, Rio de Janeiro, Brazil; 15 Wellcome-Wolfson Institute for Experimental Medicine, Queens University Belfast and Regional Intensive Care Unit, Belfast Health and Social Care Trust, Belfast, UK; 16 Department of Anesthesiology and Critical Care, Sorbonne University, GRC 29, AP-HP, DMU DREAM, Pitié-Salpêtrière Hospital, Paris, France

**Keywords:** Systematic review protocol, Acute respiratory distress syndrome, Preclinical, Subphenotypes

## Abstract

**Background:**

Subphenotypes were recently reported within clinical acute respiratory distress syndrome (ARDS), with distinct outcomes and therapeutic responses. Experimental models have long been used to mimic features of ARDS pathophysiology, but the presence of distinct subphenotypes among preclinical ARDS remains unknown. This review will investigate whether: 1) subphenotypes can be identified among preclinical ARDS models; 2) such subphenotypes can identify some responsive traits.

**Methods:**

We will include comparative preclinical (in vivo and ex vivo) ARDS studies published between 2009 and 2019 in which pre-specified therapies were assessed (interleukin (IL)-10, IL-2, stem cells, beta-agonists, corticosteroids, fibroblast growth factors, modulators of the receptor for advanced glycation end-products pathway, anticoagulants, and halogenated agents) and outcomes compared to a control condition. The primary outcome will be a composite of the four key features of preclinical ARDS as per the American Thoracic Society consensus conference (histologic evidence of lung injury, altered alveolar-capillary barrier, lung inflammatory response, and physiological dysfunction). Secondary outcomes will include the single components of the primary composite outcome, net alveolar fluid clearance, and death. MEDLINE, Embase, and Cochrane databases will be searched electronically and data from eligible studies will be extracted, pooled, and analyzed using random-effects models. Individual study reporting will be assessed according to the Animal Research: Reporting of In Vivo Experiments guidelines. Meta-regressions will be performed to identify subphenotypes prior to comparing outcomes across subphenotypes and treatment effects.

**Discussion:**

This study will inform on the presence and underlying pathophysiological features of subphenotypes among preclinical models of ARDS and should help to determine whether sufficient evidence exists to perform preclinical trials of subphenotype-targeted therapies, prior to potential clinical translation.

**Systematic review registration:**

PROSPERO (ID: CRD42019157236).

## Background

The acute respiratory distress syndrome (ARDS) is a life-threatening clinical syndrome of rapid-onset pulmonary failure characterized by a dysregulated inflammatory response that results in severe respiratory failure and the need for mechanical ventilation [1]. Although the prevalence of ARDS is low (1–5/10,000 inhabitants), its incidence is high in critically ill patients, as the syndrome is present in approximately 10% of patients upon admission to the intensive care unit (ICU) and in 24% of ICU patients under mechanical ventilation support [2]. Despite decades of research, mortality due to ARDS remains high, with significant long-term comorbidity and reduced quality of life in survivors [2]. Various pharmacotherapeutic agents have failed to improve ARDS outcomes and current treatment strategy is largely supportive, based on optimized ventilatory settings [1]. A key reason for the lack of specific pharmacological therapy is likely due to the heterogeneity within ARDS, which is diagnosed by a constellation of signs as defined by the Berlin criteria [3].

The clinical and biological heterogeneity within syndromes such as sepsis ARDS makes it essential to identify more homogeneous subphenotypes when investigating potential therapies [4, 5]. There has been recent recognition of distinct subphenotypes (on the basis of clinical/biochemical variables, natural history, disease manifestation, and/or treatment response without any implication about mechanism) and endotypes (defined by a distinct functional/pathobiological mechanism) within patients with ARDS [4, 6, 7]. Therefore, while benefit has been demonstrated in numerous preclinical studies for many candidate therapies, most have failed to translate to improved patient outcomes in randomized clinical trials (RCTs), suggesting that the appropriate subset of patients to target with the novel therapies may not have been correctly identified [8].

The retrospective analysis of data from two large multicenter RCTs (the “ventilation with lower tidal volumes as compared with traditional tidal volumes for acute lung injury and the acute respiratory distress syndrome [ARMA] and “assessment of low tidal volume and elevated end-expiratory volume to obviate lung injury” [ALVEOLI] trials) [9] has first identified at least two ARDS subphenotypes within clinical ARDS which were differentiated by the presence of shock, metabolic acidosis and a higher inflammatory status (interleukin (IL)-6 and soluble tumor necrosis factor receptor (sTNFr)-1). These two phenotypes are sometimes referred to as “endotypes” because they seem defined by distinct degrees of inflammation, but their underlying mechanisms remain largely unknown. However, these subphenotypes were stable over the first 3 days of enrolment in both RCTs, suggesting that they are linked to true biological processes. The hyper-inflammatory subphenotype demonstrated significantly worse outcomes when compared to the hypo-inflammatory subphenotype, with higher mortality and lesser ventilator-free and organ failure-free days. The analysis of plasma biomarkers in patients with ARDS previously enrolled in the MARS prospective cohort also identified two biological subphenotypes, which were very similar to those found earlier and were again associated with distinct mortality rates [10]. In the ALVEOLI trial, where a lower positive end-expiratory pressure (PEEP) strategy was compared to a higher PEEP strategy, the two subphenotypes demonstrated a distinct response in terms of major outcomes, suggesting the potential for using this subphenotypic classification to identify therapy responsive traits. This hypothesis was further confirmed through secondary analysis of the fluids and catheters treatment trial (FACTT) in which the two inflammatory subphenotypes had distinct effects of the randomly assigned fluid management strategy on 90-day mortality [11]. The potential for the inflammation-based subphenotypic classification was again demonstrated in a secondary analysis of the hydroxymethylglutaryl-CoA reductase inhibition with simvastatin in acute lung injury to reduce pulmonary dysfunction–2 (HARP-2) trial, a multicenter trial investigating the potential of simvastatin as an anti-inflammatory therapy for ARDS. Subphenotyping based on the hypo- versus hyper-inflammatory profile identified a therapy response trait as evidenced by the improved survival of patients classified as hyper-inflammatory and randomized to simvastatin [12].

Translation of basic research findings to clinical practice remains daunting because of the heterogeneity and complexity of ARDS [13]. A substantial number of preclinical models of experimental lung injury have been used to investigate mechanisms of lung injury and test novel therapies [14–16]. Although none of these experimental models fully reproduces all features of human lung injury, most of them are based on the reproduction, in animals, of known risk factors for ARDS, such as pulmonary or extrapulmonary sepsis, lipid embolism such as when secondary to bone fracture, and acid aspiration, among other clinical risks [14, 15, 17]. Recently, more translational models of acute lung injury have been used, such as the ex vivo human lung preparation [18–21]. Although the nature of the insult leading to acute lung injury in preclinical models grossly influences one or more features of human ARDS, the presence of subphenotypes among experimental models of ARDS has never been systematically assessed to date.

### Study question

We hypothesize that ARDS subphenotypes may exist among preclinical ARDS studies and that they may have distinct treatment response to select therapies.

To test this hypothesis, we designed a systematic review and meta-analysis of controlled preclinical trials in experimental, − in vivo (animal) and ex vivo (human lung preparation) -, models of ARDS. The primary goal of the review is to compare the key features of preclinical ARDS, as defined by the American Thoracic Society (ATS) consensus conference (histologic evidence of lung injury, altered alveolar-capillary barrier, lung inflammatory response, and physiological dysfunction) [15], in lung-injured experimental groups treated or not with any of some prespecified candidate therapies.

## Methods

### Protocol and registration

The systematic review and meta-analysis protocol originated from discussions between our team of translational research scientists (AC, JA, RB, DM, VS, JMC, MJ), information system specialists (NPD, MDC), a statistical and methodologist (BP), and the European Society of Intensive Care Medicine (ESICM) Translational Biology Group of the Acute Respiratory Failure section. The protocol was registered on PROSPERO (ID: CRD42019157236) in December 2019.

### Study eligibility criteria

We will include controlled comparative studies (randomized and non-randomized) of preclinical acute lung injury/ARDS published from 2009 to 2019 that evaluate the efficacy and safety outcomes of a number of predefined therapies (stem cells, fibroblast growth factors, IL-2, IL-10, beta-agonists, corticosteroids, modulators of the receptor for advanced glycation end-products (RAGE) pathway, anticoagulants, and halogenated agents). In order to collect sufficient data to test our hypotheses, it was decided a priori to include only studies in which at least two features, out of the four key features of preclinical ARDS proposed by the 2011 Official ATS Workshop Report [15], are assessed.

### Preclinical model eligibility criteria

We will include preclinical in vivo animal and ex vivo human models of experimentally induced acute lung injury that mimic at least some aspects of the pathophysiology of humans with ARDS according to the ATS consensus criteria [15]. The inclusion of a wide range of acute lung injury models should enhance the generalizability of our study findings. Acute lung injury in experimental models may be induced by several methods (Table 1). These include intravenous, intratracheal or intraperitoneal administration of bacteria or endotoxin, induction of injury by the ventilator (ventilator-induced lung injury), chemical agents (oleic acid, hydrochloric acid), trauma, shock (for example hemorrhagic or induced by sepsis), or remote organ injury (for example pancreatitis, ischemia-reperfusion). We will exclude neonatal animal models of acute lung injury because the mechanisms of disease, and efficacy of treatments, are likely to be different in this specific setting.

**Table 1:** Preclinical models of acute respiratory distress syndrome included in the review

Oleic acid	
Lipopolysaccharides	
Acid aspiration	
Hyperoxia	
Saline lavage	
Pulmonary ischemia/reperfusion	
Nonpulmonary ischemia/reperfusion	
Intravenous bacteria	
Intrapulmonary bacteria	
Peritonitis	
Cecal ligation and puncture	
Trauma/Shock	
Mechanical ventilation (ventilator-induced lung injury)	
Chemical or chemotherapeutic injury (such as detergents)	
Pancreatitis	
Burns	

### Interventions

Preclinical intervention groups will include animals and ex vivo human lungs from studies that examine the therapeutic use of stem cells (and their derived microvesicles), fibroblast growth factors, IL-2, IL-10, beta-agonists, corticosteroids, RAGE modulators, anticoagulants, and halogenated agents such as isoflurane, sevoflurane, and desflurane (Table 2). These interventions have been selected a priori because they are the main areas of interest of our research groups [22–30]. In addition to studies of therapies that are administered after lung injury is modeled, experiments using pretreatment will also be included since they are clinically relevant for the prevention of ARDS. Moreover, studies using a co-treatment or multiple injurious hits will be included.

**Table 2:** Predefined list of select therapies included in the meta-analysis

Class	Example of therapies
Interleukins	IL-10, IL-2
Cell therapy	MSCs, MSC-derived microvesicles
Adrenergic beta-agonists	Salbutamol, albuterol, sultanol, proventil
Growing factors	Fibroblast growing factors, keratinocyte growth factor, endothelial growth factor
RAGE modulators	Anti-RAGE antibody, recombinant soluble RAGE, gene knockout, endogenous secretory RAGE
Corticosteroids	Glucocorticoids, mineralocorticoids, 3-oxo steroid, 17-ketosteroids, domolene, cortifan, epicortisol, komed-hc, heb-cort, prednisone, encortone, panasol, deltacortisone, decortisyl, decortin, predeltin, orasone, prednidib, prednisone, dacortin, cortancyl, solupred, deltasone, pronisone, sterapred, encorton, panafcort, delta-dome, dehydrocortisone, prednisonum, lisacort, ultracorten, winpred, metacortandracin, meticorten, prednisolone, predate, prednisolone, di-adreson f, delta-1-hydrocortisone, prednisolonum, sterane, meti derm, metacortandralone, predonine
Anticoagulant agents	Heparin, antithrombin, plasminogen activator, fibrinolytic agents, tissue factor pathway inhibitor, lipoprotein-associated coagulation inhibitor, thrombin-thrombomodulin complex, thrombin receptor, activated protein C, drotrecogin alfa activated
Halogenated agents	Sevoflurane, desflurane, isoflurane

*IL* interleukin; *MSCs* mesenchymal stem (stromal) cells; *RAGE* receptor for advanced glycation end-products

### Comparison

The preclinical comparison group will include data from studies that have had experimental acute lung injury induced but have not been administered any of the prespecified therapies listed in the previous paragraph. This will allow to perform effect size calculations in our meta-analysis to examine how efficacious and safe these therapies are in preclinical ARDS in general, and in preclinical ARDS subphenotypes in particular. In addition, we will use other control groups, such as healthy or sham-injured controls, to examine the severity of preclinical ARDS.

### Preclinical primary endpoint

The primary endpoint is a composite of the four key features that define preclinical ARDS, with specific measurements defined by the consensus criteria proposed by the ATS [15].

These include histological evidence of tissue injury (usually based on the lung injury score), alteration of the alveolar-capillary barrier (for example increased concentration of high molecular weight proteins in bronchoalveolar fluid), measures of the inflammatory response in the lung (for example pulmonary leukocytes or neutrophils, cytokines), and measures of physiological dysfunction such as the alveolar-arterial gradient of oxygen concentration and the partial pressure of arterial oxygen (PaO_2_) to fraction of inspired oxygen (FiO_2_) ratio. These quantitative features and measurements are summarized in Table 3. We will focus on the “very relevant” measurements defined by the consensus criteria [15].

**Table 3:** Acute lung injury features and measurements

Features	Relevant measurements
Histological evidence of lung injury	Accumulation of neutrophils in the alveolar/interstitial space Formation of hyaline membranes Proteinaceous debris in alveolar space Proteinaceous debris in alveolar space Thickening of the alveolar wall Injury by a standardized histology score Evidence of hemorrhage Areas of atelectasis Gross macroscopic changes such as discoloration of the lungs
Alteration of the alveolar capillary barrier	Increased extravascular lung water content Accumulation of protein/tracer in airspaces/extravascular space Total BAL protein concentration BAL concentration of high molecular weight proteins Vascular filtration coefficient Translocation of a protein from the airspaces into plasma Increased lung lymph flow, lymph protein concentration
Inflammation	BAL total neutrophil counts Lung MPO activity Concentrations of cytokines and chemokines in lung tissue, in BAL fluid, and/or in plasma
Physiological dysfunction	Hypoxemia Increased A-a oxygen difference

*BAL* bronchoalveolar lavage; *MPO* myeloperoxidase

Because the assessment of our composite endpoint must reflect that the timing of experimental induction of ARDS may vary across studies, the primary endpoint will be analyzed in categories of time from the induction of ARDS of less than 6 h, between 6 and 24 h, and greater than 24 h. These time points for measurement were selected since the development of inflammation and acute lung injury occurs over time and contributes to death and morbidity in this population [2].

### Preclinical secondary endpoints

Secondary endpoints will include death, net alveolar fluid clearance, and each individual component of the primary composite endpoint. The same categories of time will be used for primary and secondary endpoints. Where reported, the occurrence of adverse events will also be documented for each included study. For animal models that use infectious induction of acute lung injury, we will describe pathogen clearance (for example number of bacterial colony-forming units).

### Information sources

Search strategies and equation of searching have been developed and tested with the help of two librarians using the Peer Review of Electronic Search Strategies (PRESS) template (see Additional file 1 for the representative search strategy) [31]. We will search on MEDLINE, Embase, and Cochrane databases. Search strategies will use a combination of controlled vocabulary (for example corticoids, mesenchymal stem cells, acute lung injury, respiratory distress syndrome) and keywords (for example mesenchymal stromal cells, acute lung injury, acute/adult respiratory distress syndrome). Vocabulary and syntax will be adjusted across the databases. Two recently published animal filters [32, 33], validated for PubMed/MEDLINE and Embase and amended slightly, will be used to increase relevancy. These filters will be adjusted for use in the other databases where a validated filter is unavailable. Language will be restricted to English studies and no studies published before 2009 will be included. The bibliographies of included studies and relevant reviews will also be hand searched for further preclinical studies.

### Study selection

The titles and abstracts of search results will be screened independently by two investigators (AC, MJ). The full text of all potentially eligible studies will be retrieved and reviewed for eligibility. Disagreements between reviewers will be resolved by consensus or by a third member of the systematic review team (JA). Reasons for exclusion of potentially eligible studies will be recorded to enable a transparent selection process and to be in accordance with the Preferred Reporting Items for Systematic Reviews and Meta-Analyses (PRISMA) guidelines developed for proper reporting of clinical systematic reviews [34]. The Preferred Reporting Items for Systematic Reviews and Meta-Analyses Protocols (PRISMA-P) checklist for this review protocol is available as Additional file 2.

### Data collection and process and data items

Data will be extracted by four members (AC, JA, RZ, MJ) of the research team and collected into standardized, pilot-tested forms using REDCap electronic data capture tools hosted at CHU Clermont-Ferrand [35]. REDCap (Research Electronic Data Capture) is a secure, web-based application designed to support data capture for research studies, providing: (1) an intuitive interface for validated data entry; (2) audit trails for tracking data manipulation and export procedures; (3) automated export procedures for seamless data downloads to common statistical packages; and (4) procedures for importing data from external sources. Specific data elements are listed in Table 4.

**Table 4:** Data collection elements

Categories	Main items
Study characteristics	DOI, Num ID (specific number ID for the purpose of the meta-analysis), title, author, year, country and journal of publication, country of study, mono/multicentric
Study population (experimental model)	Animal species or ex vivo model, age, gender, strain, weight, and comorbidity
Type of acute lung injury model	As defined in Table 1, with timing
Intervention	Type (see Table 2), number, timing, mode of administration, dose
Co-intervention	Resuscitation fluids, antibiotics, mechanical ventilation parameters, mode of anesthesia, drugs, etc.
Preclinical endpoints	As defined in Table 3
Risk of bias assessments (Cochrane risk of bias tool)	Randomization, allocation, blinding and completeness of follow-up
Quality of reporting of individual preclinical studies	ARRIVE (Animal Research: Reporting of In Vivo Experiments) guidelines elements

*DOI* digital object identifier

### Assessment of risk of bias

The risk of bias will be evaluated by two reviewers (AC, MJ) for each included preclinical study. Since no validated and standardized risk of bias checklist exists for preclinical studies, we will describe the biases of the included studies using the Cochrane risk of bias assessment [36] and the Risk Of Bias In Nonrandomized Studies - of Interventions (ROBINS-I) [37] tools. Items in this tool include assessments for concealment of allocation, random sequence generation, blinding of personnel and the endpoint measurements, and completeness of endpoint reporting. Each bias criterion will be assigned a value of low, high, or unclear risk of bias for each included study [36].

### Assessment of construct validity and external validity

We will also record features that will facilitate judgements of construct validity and external validity [38]. Construct validity in preclinical research concerns the extent to which an experimental system accurately models a clinical entity. These will include: age, gender, weight, species, and strain of animal; presence of comorbidities; type of acute lung injury model; timing, dose and mode of administration of treatment; and use of co-interventions (for example fluid resuscitation, type of anesthesia, use of antibiotics for infectious acute lung injury models) (Table 5). External validity in preclinical research concerns the extent to which cause and effect relationships holdup under varied conditions [39], such as the use of a multicenter preclinical design.

**Table 5:** Elements of construct validity and external validity

Category	Items
Animal species and strain (for mice)	Human lung preparation, mouse, rat, sheep, pig, rabbit, dog or others
Animal age	Young, middle-aged, and mature, depending on species
Animal gender	Male, female, both
Comorbidity	Yes or no, type
Model of acute lung injury	As defined in Table 1
Severity of lung injury	Lung injury score
Intervention type	The drug, mode of administration and dose
Timing of intervention regarding to acute lung injury induction	Before lung injury, 0 to 1, 1 to 6, and > 6 h after lung injury
Type of control	Injured, non-injured
Use of co-interventions	Resuscitation fluids, use of antibiotics, type of anesthesia, mechanical ventilation strategy
Number of study centers	Single versus multi-center

### Description of reporting

We will describe the quality of reporting of the included preclinical studies using the elements of the Animal Research: Reporting of In Vivo Experiments (ARRIVE) guidelines. The ARRIVE guidelines were developed to enhance the transparent and comprehensive reporting of research methods and results for in vivo animal experiments [40].

### Ethics and dissemination

This study does not require ethical approval. Data collected through this systematic review will be managed by the ESICM Translational Biology Group and made available upon reasonable request. The results of this systematic review will be presented at relevant conferences and published in a peer-reviewed journal.

### Data analysis

Statistical analyses will be performed using Stata (version 15; StataCorp, College Station, US), Comprehensive Meta-Analysis (version 2; Biostat, Englewood, NJ), and Mplus [41] softwares. For descriptive analyses, data will be presented as mean and standard deviation or median and interquartile range, according to statistical distribution. To address the non-independence of data due to study effect, random-effects models will be performed rather than fixed-effect as certain experimental parameters can have wide variation. The dichotomous endpoints from each included study will be pooled and described as relative risks (RR) and 95% confidence intervals incorporating a random effects modeling approach with the use of forest plots for presentation of the data [42]. Continuous endpoints will be pooled using the ratio of weighted means method with inverse variance random effects modeling and Hedges’s standardized mean differences (SMD) [43]. When necessary, the RRs reported in individual studies will be converted into SMD [44]. The statistical heterogeneity of included preclinical studies will be measured using the I^2^ [45], which will be used to measure heterogeneity with 25, 50 and 75% indicating low, moderate and high heterogeneity, respectively. Egger’s test and funnel plots will be used to assess publication bias. In the absence of bias, studies will be distributed evenly around the mean effect size because of random sampling error.

Then, meta-regression will be conducted, with covariates determined according to the aforementioned analyses and to clinical and/or biological relevance, and methodological issues such as the categories of time from ARDS induction. Particular attention will be paid to the study of multicollinearity and interactions between covariates 1) studying the relationships between the covariates and 2) evaluating the impact of adding or removing variables to or from the multivariable model. Whenever possible in appropriate data, latent class analyses will be carried out to identify subphenotypes within experimental models of ARDS, associated with distinct outcomes and differential responses to therapy. Statistical analyses will be conducted including all controlled comparative studies, with the type of study considered as stratification variable (randomized and non-randomized).

When the number of studies and sample size will permit, analyses will be performed by study type. Several subgroup analyses to examine preclinical heterogeneity will be conducted on the primary endpoint. These analyses will include “clinical”/biological features (such as the type of experimental model; animal age, gender, weight, species, and strain; presence of comorbidities; cause of experimental ARDS; severity of the ARDS model; type of tested therapies (Table 2); type of controls; type of anesthesia; use of co-interventions, antibiotics, and mechanical ventilation settings; multiple-hit versus single-hit model of ARDS) and methodological features (such as single versus multi-center study, presence of an a priori sample size calculation). These subgroup analyses will be exploratory and the results will be interpreted with caution. In addition, subphenotypes that may be identified among preclinical studies will be compared to those already described in Humans (and those that could be identified in the future) using the same set of variables than in patients such as plasma levels of some proinflammatory cytokines, when applicable [6, 7].

Sensitivity analyses will be conducted to assess how including and excluding studies influences the results. More precisely, sensitivity analyses will be performed to measure the impact of high heterogeneity or a methodological quality estimated too low, and risk of bias assessments. In addition, sensitivity analyses will be performed, when possible, to assess the effects of the nature, sequence, and timing of all the injurious hits that are used in studies based on multiple-hit models of experimental ARDS.

## Discussion

The results of this systematic review and meta-analysis will inform translational and clinical scientists, researchers, and clinicians internationally regarding the existing preclinical evidence for subphenotypes among preclinical, in vivo or ex vivo models of ARDS. Such data will be crucial to confirm the potential of personalized ARDS medicine [6, 8, 46], i.e. the promise to deliver the right -targeted- treatment to the right subject. These data will also inform on the potential value of testing candidate therapies (including therapies that have already been tested in previous studies) that target distinct functional and/or pathobiological processes (such as lung epithelial injury and alveolar inflammation [9, 47]) in select experimental models that might best mimic ARDS subphenotypes described in patients to date [6]. For example, although it seems plausible that tracheal instillation with hydrochloric acid causes prominent injury to the alveolar epithelium and live bacteria (such as *Streptococcus pneumoniae*) causes major alveolar inflammation profiles, it remains unknown whether these models might have reliable value to represent clinical subphenotypes of higher inflammation and of higher degree of lung epithelial injury, respectively [6].

Our review is timely since there is an increasing amount of research dedicated to the identification of ARDS subphenotypes, their underlying mechanisms, and their potential responsive treats [4, 7, 9–12, 48–52]. The results from this study should therefore be useful in order to generate hypotheses on whether some therapies (see Table 2) might differentially benefit to some preclinical subphenotypes, as compared to others. In case of positive results, this might open new perspectives on preclinical precision therapies, with the ultimate goal of future clinical translation. However, the predefined, limited number of therapies investigated through our review (see Table 2) is a limitation that should be acknowledged; this limited number reflects both our specific research topics of interest and a balance between scientific validity and feasibility. Our results will inform on whether the nature of the lung injurious hit used in published experimental models of ARDS could identify subphenotypes with distinct degrees of severity and treatment effects. Yet, we might not be able to distinguish such subphenotypes using individual subject data analysis in a given experimental model, such as recently reported in a secondary analysis of a murine cecal ligation and puncture sepsis model, with one subphenotype characterized by faster deterioration and more severe inflammation than the other and distinct responses to some interventions (immediate versus delayed antibiotics and fluids) [53].

In a broader perspective, we also hope this review will increase our knowledge on, and identify challenges and barriers related to, the conduct of preclinical ARDS studies. Ultimately, it will also inform and enrich future preclinical and clinical precision, subphenotype-based, research that should aid the translation of novel therapies for ARDS.

## Supplementary information

**Additional file 1.** Representative Search Strategy.

**Additional file 2.** Preferred reporting items for systematic review and meta-analysis protocols (PRISMA-P) checklist.

## Data Availability

Data collected through this systematic review will be managed by the ESICM Translational Biology Group and made available upon reasonable request.

## Abbreviations

ALVEOLI: Assessment of low tidal volume and elevated end-expiratory volume to obviate Lung injury
ARDS: Acute respiratory distress syndrome
ARMA: Ventilation with lower tidal volumes as compared with traditional tidal volumes for acute lung injury and the acute respiratory distress syndrome
ARRIVE: Animal research: reporting of in vivo experiments
ATS: American thoracic society
ESICM: European society of intensive care medicine
FACTT: Fluids and catheters treatment trial
FiO_2_: Fraction of inspired oxygen
HARP-2: Hydroxymethylglutaryl-CoA reductase inhibition with simvastatin in acute lung injury to reduce pulmonary dysfunction–2
ICU: Intensive care unit
IL: Interleukin
PaO_2_: Partial pressure of arterial oxygen
PEEP: Positive end-expiratory pressure
PRESS: Peer review of electronic search strategies
PRISMA: Preferred reporting items for systematic reviews and meta-analyses
PRISMA-P: Preferred reporting items for systematic reviews and meta-analyses protocols
REDCAP: Research electronic data capture
RAGE: Receptor for advanced glycation end-products
RCT: Randomized clinical trial
ROBINS-I: Risk of bias in nonrandomized studies - of interventions
RR: Relative risk
SMD: Standardized mean differences
sTNFr-1: Soluble tumor necrosis factor receptor 1
