# Supplementary material for: Acute respiratory distress syndrome subphenotypes and therapy responsive traits among preclinical models: protocol for a systematic review and meta-analysis
Source: Respir Res. 2020 Apr 7;21:81. doi: 10.1186/s12931-020-01337-9 (PMC7137453; doi:10.1186/s12931-020-01337-9)
Supplement: Supplementary file 1 — Additional file 1. Representative Search Strategy. [file 12931_2020_1337_MOESM1_ESM.docx]

**Additional file 1**

**Representative Search Strategy. Databases: MEDLINE, EMBASE, COCHRANE.**

**Period: from 2009 to 2019.**

**Database: MEDLINE <2009 to 2019>**

("lung injury"[MH:noexp] OR "lung injury"[TW] OR "lung injuries"[TW] OR "pulmonary injuries"[TW] OR "injury, pulmonary"[TW] OR "injury, lung"[TW] OR "pulmonary injury"[TW] OR "Injury of lung"[TW] OR “lung diseases"[MH:noexp] OR "acute lung injury"[MH]OR "Ventilator-Induced lung injury"[MH:noexp] OR ("Ventilators, Mechanical"[Mesh:NoExp] AND injury[TW]) OR "respiratory distress syndrome, adult"[MH]OR "congestive atelectasis"[TW] OR "traumatic wet lung"[TW] OR "adult respiratory distress syndrome"[TW] OR "respiratory distress syndrome adult"[TW] OR "acquired respiratory distress syndrome"[TW] OR "non-cardiogenic pulmonary edema"[TW] OR "lung shock"[TW] OR "adult rds"[TW] OR "respiratory distress syndrome acute"[TW] OR "ards"[TW] OR "pulmonary insufficiency following trauma"[TW] OR "human ards"[TW] OR "acute respiratory distress syndrome (ards)"[TW] OR "danang lung"[TW] OR "respiratory distress syndrome acute"[TW] OR "acute respiratory distress syndrome"[TW] OR "non-cardiogenic pulmonary oedema"[TW] OR "respiratory distress; syndrome, adult"[TW] OR "shock lung"[TW] OR "adult hyaline membrane disease"[TW] OR "respiratory distress syndrome adult"[TW] OR "post-traumatic pulmonary insufficiency"[TW] OR "pulmonary capillary leak syndrome"[TW] OR "pneumonia"[MH]OR "inflammatory lung disease"[TW] OR "pneumonitis"[TW] OR "lung inflammation"[TW] OR "pneumonia"[TW] OR "bronchopneumonia"[TW] OR "inflammation lung"[TW] OR "pneumonitides"[TW] OR "inflammation pulmonary"[TW] OR "pneumonias"[TW] OR "pulmonitis"[TW] OR "pulmonary inflammations"[TW] OR "lung inflammations"[TW] OR "pulmonary inflammation"[TW] OR "pulmonary edema"[MH]OR "edema pulmonary"[TW] OR "wet lung"[TW] OR "pulmonary edema"[TW] OR "lungs wet"[TW] OR "edema lung"[TW] OR "wet lungs"[TW] OR "pulmonary oedemas"[TW] OR "lung edema"[TW] OR "pulmonary edemas"[TW] OR "pulmonary oedema"[TW] OR "lung wet"[TW] OR "lung oedema"[TW] OR "respiratory insufficiency"[MH:noexp] OR "ventilatory depression"[TW] OR "respiratory insufficiency"[TW] OR "insufficiency respiratory"[TW] OR "insufficiency pulmonary"[TW] OR "respiratory impairment"[TW] OR "respiratory failure"[TW] OR "pulmonary incompetence"[TW])

AND

("Interleukin-10"[MH]OR "cytokine synthesis inhibiting factor"[TW] OR "il10"[TW] OR "il-10"[TW] OR "cytokine synthesis inhibitory factor"[TW] OR "tgif"[TW] OR "interleukin-10"[TW] OR "t-cell growth inhibitory factor"[TW] OR "interleukin 10"[TW] OR "csif"[TW] OR "Interleukin-2"[MH]OR "ru49637"[TW] OR "thymocyte stimulating factor"[TW] OR "interleukin 2"[TW] OR "mitogenic factor, lymphocyte"[TW] OR "t cell growth factor"[TW] OR "il-2"[TW] OR "t-cell stimulating factor"[TW] OR "ru 49637"[TW] OR "tcgf"[TW] OR "lymphocyte mitogenic factor"[TW] OR "interleukin ii"[TW] OR "interleukine 2"[TW] OR "epidermal thymocyte activating factor"[TW] OR "ru-49637"[TW] OR "t-cell growth factor"[TW] OR "interleukin-2"[TW] OR "mitogenic factor"[TW] OR "etaf"[TW] OR "t cell stimulating factor"[TW] OR "stem cells"[MH]OR "progenitor cell"[TW] OR "stem cell"[TW] OR "colony forming unit"[TW] OR "colony-forming units"[TW] OR "cell, stem"[TW] OR "hematopoietic progenitor cell"[TW] OR "colony forming units"[TW] OR "cells, mother"[TW] OR "cord blood"[TW] OR "mother cells"[TW] OR "hematopoietic progenitor cells"[TW] OR "mother cell"[TW] OR "cells, stem"[TW] OR "colony-forming unit"[TW] OR "progenitor cells"[TW] OR "stem cells"[TW] OR "cells, progenitor"[TW] OR "mesenchymal stromal cells"[TW] OR "whartons jelly cells"[TW] OR "cells, mesenchymal progenitor"[TW] OR "marrow stromal cells"[TW] OR "marrow stromal cell"[TW]OR "mesenchymal stromal cell"[TW] OR "wharton jelly cells"[TW] OR "mesenchymal progenitor cell"[TW] OR "wharton's jelly cell"[TW] OR "wharton's jelly cells"[TW] OR "cells, mesenchymal stem"[TW] OR "mesenchymal progenitor cells"[TW] OR "Microvesicles"[TW]OR "Secretome"[TW] OR"albuterol"[MH]OR "18559-94-9"[TW] OR "albuterol"[TW] OR "qf8svz843e"[TW] OR "salbutamol"[TW] OR "sultanol"[TW] OR "ventolin"[TW] OR "proventil"[TW] OR "β₂-agonists"[TW] OR "adrenergic beta-agonists"[MH]OR "betamimetics"[TW] OR "beta-adrenergic agonists"[TW] OR "adrenergic beta agonist"[TW] OR "adrenergic beta-receptor agonists"[TW] OR "beta-adrenergic agonist"[TW] OR "adrenergic beta-receptor agonist"[TW] OR "adrenergic beta receptor agonist"[TW] OR "beta adrenergic agonists"[TW] OR "beta-adrenergic receptor agonists"[TW] OR "beta adrenergic receptor agonists"[TW] OR "beta-adrenergic receptor agonist"[TW] OR "adrenergic beta agonists"[TW] OR "adrenergic beta-agonists"[TW] OR "adrenergic beta receptor agonists"[TW] OR "beta adrenergic receptor agonist"[TW] OR "adrenergic beta-agonist"[TW] OR "beta adrenergic agonist"[TW] OR "agonists, beta-adrenergic"[TW] OR "Adrenergic beta-2 receptor agonists"[TW] OR "Beta2 adrenergic agonist"[TW] OR "Beta-2-agonist"[TW] OR "Beta-adrenoceptor agonists"[TW] OR "keratinocyte growth factor receptor"[Supplementary Concept] OR "keratinocyte growth factor"[TW] OR "KGF"[TW] OR "fibroblast growth factors"[MH] OR "growth factor, fibroblast"[TW] OR "dna synthesis factor"[TW] OR "fgf family"[TW] OR "growth factors, fibroblast"[TW] OR "endothelial cell growth factor"[TW] OR "fibroblast growth regulatory factor"[TW] OR "ecgf"[TW] OR "fibroblast growth factors"[TW] OR "fibroblast growth factor"[TW] OR "Receptor for Advanced Glycation End Products"[MH] OR "advanced glycosylation end-product receptor"[TW] OR "amphoterin receptor"[TW] OR "receptor for advanced glycation end products"[TW] OR "advanced glycosylation end product receptor"[TW] OR "advanced glycosylation end product specific receptor"[TW] OR "age receptor"[TW] OR "advanced glycosylation end product-specific receptor"[TW] OR "ager protein"[TW] OR "RAGE"[TW] OR "sRAGE"[TW] OR "esRAGE"[TW] OR "FPS-ZM1"[TW] OR "PF-04494700"[TW] OR "corticoids"[TW] OR "Corticosteroids"[TW] OR ("steroids"[MH] AND "anti-inflammatory agents"[PA])OR "adrenal cortex hormones"[Mesh:NoExp] OR "adrenal cortex hormones"[TW] OR "17-Ketosteroids"[MH]OR "ketosteroids"[TW] OR "oxosteroids"[TW] OR "glucocorticoids"[MH]OR "glucocorticoid"[TW] OR "glucorticoid effects"[TW] OR "glucocorticoids"[TW] OR "hydroxycorticosteroids"[MH]OR "hydroxycorticosteroids"[TW] OR "hydroxycorticosteroid"[TW] OR "hydrocortisone"[MH]OR "11-epicortisol"[TW] OR "50-23-7"[TW] OR "domolene"[TW] OR "cortisol"[TW] OR "cortifan"[TW] OR "wi4x0x7bpj"[TW] OR "barseb-hc"[TW] OR "epicortisol"[TW] OR "epicortisol"[TW] OR "komed-hc"[TW] OR "rectoid"[TW] OR "barseb hc"[TW] OR "(11beta)-11,17,21-trihydroxypregn-4-ene-3,20-dione"[TW] OR "heb-cort"[TW] OR "cortril"[TW] OR "hydrocortisone"[TW] OR "mineralocorticoids"[MH] OR "mineralocorticoids"[TW] OR "mineralocorticoid hormone"[TW] OR "mineralocorticoid effects"[TW] OR "prednisone"[MH]OR "1,2-dehydrocortisone"[TW] OR "encortone"[TW] OR "1,2-dehydrocortisone"[TW] OR "panasol"[TW] OR "deltacortisone"[TW] OR "decortisyl"[TW] OR "delta-cortisone"[TW] OR "decortin"[TW] OR "cortan"[TW] OR "rectodelt"[TW] OR "prednicen-m"[TW] OR "liquid pred"[TW] OR "vb0r961hzt"[TW] OR ".delta.1-cortisone"[TW] OR "predeltin"[TW] OR "delta 1-cortisone"[TW] OR "orasone"[TW] OR "prednidib"[TW] OR "prednisone"[TW] OR "panafcort"[TW] OR "dacortin"[TW] OR "cortancyl"[TW] OR "pred"[TW] OR "53-03-2"[TW] OR "deltasone"[TW] OR "pronisone"[TW] OR "encorton"[TW] OR "delta-dome"[TW] OR "dehydrocortisone"[TW] OR "prednisonum"[TW] OR "lisacort"[TW] OR "sterapred"[TW] OR "ultracorten"[TW] OR "winpred"[TW] OR "metacortandracin"[TW] OR "meticorten"[TW] OR "sone"[TW] OR "prednisolone"[MH]OR "50-24-8"[TW] OR "predate"[TW] OR "prednisolone"[TW] OR "(11beta)-11,17,21-trihydroxypregna-1,4-diene-3,20-dione"[TW] OR "di adreson f"[TW] OR "deltahydrocortisone"[TW] OR "9phq9y1olm"[TW] OR ".delta.1-hydrocortisone"[TW] OR "prednisolonum"[TW] OR "sterane"[TW] OR "delta-f"[TW] OR "meti derm"[TW] OR "di-adreson-f"[TW] OR "metacortandralone"[TW] OR "delta(1)hydrocortisone"[TW] OR "predonine"[TW] OR "Inhaled sedation"[TW] OR "Halogenated agent"[TW] OR "Halogenated agents"[TW] OR "Halogenates"[TW] OR "Halogenate"[TW] OR "Volatile anesthetics"[TW] OR "Volatile agents"[TW] OR "Sevoflurane"[TW] OR "sevorane"[TW] OR "isoflurane"[MH] OR "isoflurane"[TW] OR "Desflurane"[TW] OR "anticoagulant*"[MH] OR "anticoagulant"[TW] OR "fibrinolytic agents"[MH] OR "fibrinolytic"[TW] OR "tissue factor pathway inhibitor"[TW] OR "lipoprotein-associated coagulation inhibitor"[TW] OR "Thrombin-thrombomodulin complex"[TW] OR "thrombin receptor "[TW] OR "activated protein C "[TW] OR "drotecogin alfa" [TW] OR "antithrombins"[MH] OR "antithrombin"[TW] OR "plasminogen activator"[TW] OR "heparin"[MH])

AND

(Animals[Mesh:noexp] OR "animal*"[TW] OR experimental*[TW] OR "in vivo"[TW] OR "mice"[TW] OR "rats"[TW] OR "mouse"[TW] OR "pig"[TW] OR "pigs"[TW] OR "piglet*"[TW] OR "rabbit"[TW] OR "sheep*"[TW] OR "swine*"[TW] OR "dog"[TW] OR "dogs"[TW] OR "non-human primates"[TW] OR “ex vivo"[TW])

AND

("therapy"[Subheading] OR "therapy"[TW] OR "treatment*"[TW] OR "therapeutics"[MeSH Terms] OR "therapeutic*"[TW] OR "drug therapy"[TW] OR “effect*”[title] OR "treat"[TW] OR "treating"[TW] OR "therapies"[TW] OR "treated"[TW] OR "treats"[TW] OR "pharmacology"[MH] OR "pharmacolog*"[TW] OR "pharmacotherapy"[TW])

**Database: EMBASE <2009 to 2019>**

('lung disease'/exp OR 'respiratory failure'/exp OR 'respiratory distress syndrome'/exp OR 'hyaline membrane disease'/exp)

AND

('interleukin 10'/exp OR 'il10 gene'/exp OR 'ager protein rat'/exp OR 'ager protein mouse'/exp OR 'advanced glycation end product receptor antagonist'/exp OR 'rage gene'/exp OR 'inhalational drug administration'/exp OR 'beta 2 adrenergic receptor stimulating agent'/exp OR 'thymocyte activating factor'/exp OR 'ru 49637'/exp OR 'tgif gene'/exp OR 'interleukin 2'/exp OR 'mitogenic agent'/exp OR 'stem cell'/exp OR 'colony forming unit'/exp OR 'umbilical cord blood'/exp OR 'hematopoietic stem cell'/exp OR 'mesenchymal stroma cell'/exp OR 'membrane microparticle'/exp OR 'secretome'/exp OR 'salbutamol'/exp OR 'salbutamol sulfate'/exp OR 'keratinocyte growth factor receptor'/exp OR 'fibroblast growth factor'/exp OR 'endothelial cell growth factor'/exp OR 'high mobility group b1 protein'/exp OR 'fps zm 1'/exp OR 'azeliragon'/exp OR 'corticosteroid'/exp OR '17 oxosteroid'/exp OR 'oxosteroid'/exp OR 'glucocorticoid'/exp OR 'hydroxycorticosteroid'/exp OR 'hydrocortisone'/exp OR 'mineralocorticoid'/exp OR 'prednisone'/exp OR 'dihydrocortisone'/exp OR 'sone'/exp OR 'sevoflurane'/exp OR 'prednisone acetate'/exp OR 'hydrocortisone acetate'/exp OR 'epihydrocortisone'/exp OR 'isoflurane'/exp OR 'desflurane'/exp OR 'anticoagulant agent'/exp OR 'fibrinolytic agent'/exp OR 'tissue factor pathway inhibitor'/exp OR 'thrombin receptor'/exp OR 'activated protein c'/exp OR 'antithrombin'/exp OR 'plasminogen activator'/exp OR 'heparin'/exp)

AND

('animal cell'/de OR 'animal experiment'/de OR 'animal model'/de OR 'animal tissue'/de OR 'nonhuman'/de)

AND

('drug therapy'/lnk OR 'pharmacology'/lnk OR 'therapy'/lnk)

AND

[embase]/lim NOT ([embase]/lim AND [medline]/lim)

AND

[2009-2019]/py

**Database: COCHRANE <2009 to 2019>**

**#1** ("lung injury" OR "lung injuries" OR "pulmonary injuries" OR "injury, pulmonary" OR "injury, lung" OR "pulmonary injury" OR "Injury of lung" OR "lung diseases" OR ("Ventilators, Mechanical" AND injury ) OR "respiratory distress syndrome, adult" OR "congestive atelectasis" OR "traumatic wet lung" OR "adult respiratory distress syndrome" OR "respiratory distress syndrome adult" OR "acquired respiratory distress syndrome" OR "non-cardiogenic pulmonary edema" OR "lung shock" OR "adult rds" OR "respiratory distress syndrome acute" OR "ards" OR "pulmonary insufficiency following trauma" OR "human ards" OR "acute respiratory distress syndrome (ards)" OR "danang lung" OR "respiratory distress syndrome acute" OR "acute respiratory distress syndrome" OR "non-cardiogenic pulmonary oedema" OR "respiratory distress; syndrome, adult" OR "shock lung" OR "adult hyaline membrane disease" OR "post-traumatic pulmonary insufficiency" OR "pulmonary capillary leak syndrome" OR "inflammatory lung disease" OR "pneumonitis" OR "lung inflammation" OR "pneumonia" OR "bronchopneumonia" OR "inflammation lung" OR "pneumonitides" OR "inflammation pulmonary" OR "pneumonias" OR "pulmonitis" OR "pulmonary inflammations" OR "lung inflammations" OR "pulmonary inflammation" OR "edema pulmonary" OR "wet lung" OR "pulmonary edema" OR "lungs wet" OR "edema lung" OR "wet lungs" OR "pulmonary oedemas" OR "lung edema" OR "pulmonary edemas" OR "pulmonary oedema" OR "lung wet" OR "lung oedema" OR "ventilatory depression" OR "respiratory insufficiency" OR "insufficiency respiratory" OR "insufficiency pulmonary" OR "respiratory impairment" OR "respiratory failure" OR "pulmonary incompetence" )

**#2** ("cytokine synthesis inhibiting factor" OR "il10" OR "il-10" OR "cytokine synthesis inhibitory factor" OR "tgif" OR "interleukin-10" OR "t-cell growth inhibitory factor" OR "interleukin 10" OR "csif" OR "ru49637" OR "thymocyte stimulating factor" OR "interleukin 2" OR "mitogenic factor, lymphocyte" OR "t cell growth factor" OR "il-2" OR "t-cell stimulating factor" OR "ru 49637" OR "tcgf" OR "lymphocyte mitogenic factor" OR "interleukin ii" OR "interleukine 2" OR "epidermal thymocyte activating factor" OR "ru-49637" OR "t-cell growth factor" OR "interleukin-2" OR "mitogenic factor" OR "etaf" OR "t cell stimulating factor" OR "progenitor cell" OR "stem cell" OR "colony forming unit" OR "colony-forming units" OR "cell, stem" OR "hematopoietic progenitor cell" OR "colony forming units" OR "cells, mother" OR "cord blood" OR "mother cells" OR "hematopoietic progenitor cells" OR "mother cell" OR "cells, stem" OR "colony-forming unit" OR "progenitor cells" OR "stem cells" OR "cells, progenitor" OR "mesenchymal stromal cells" OR "whartons jelly cells" OR "cells, mesenchymal progenitor" OR "marrow stromal cells" OR "marrow stromal cell" OR "mesenchymal stromal cell" OR "wharton jelly cells" OR "mesenchymal progenitor cell" OR "wharton's jelly cell" OR "wharton's jelly cells" OR "cells, mesenchymal stem" OR "mesenchymal progenitor cells" OR "Microvesicles" OR "Secretome" OR "18559-94-9" OR "albuterol" OR "qf8svz843e" OR "salbutamol" OR "sultanol" OR "ventolin" OR "proventil" OR "β₂-agonists" OR "betamimetics" OR "beta-adrenergic agonists" OR "adrenergic beta agonist" OR "adrenergic beta-receptor agonists" OR "beta-adrenergic agonist" OR "adrenergic beta-receptor agonist" OR "adrenergic beta receptor agonist" OR "beta adrenergic agonists" OR "beta-adrenergic receptor agonists" OR "beta adrenergic receptor agonists" OR "beta-adrenergic receptor agonist" OR "adrenergic beta agonists" OR "adrenergic beta-agonists" OR "adrenergic beta receptor agonists" OR "beta adrenergic receptor agonist" OR "adrenergic beta-agonist" OR "beta adrenergic agonist" OR "agonists, beta-adrenergic" OR "Adrenergic beta-2 receptor agonists" OR "Beta2 adrenergic agonist" OR "Beta-2-agonist" OR "Beta-adrenoceptor agonists" OR "keratinocyte growth factor" OR "KGF" OR "growth factor, fibroblast" OR "dna synthesis factor" OR "fgf family" OR "growth factors, fibroblast" OR "endothelial cell growth factor" OR "fibroblast growth regulatory factor" OR "ecgf" OR "fibroblast growth factors" OR "fibroblast growth factor" OR "advanced glycosylation end-product receptor" OR "amphoterin receptor" OR "receptor for advanced glycation end products" OR "advanced glycosylation end product receptor" OR "advanced glycosylation end product specific receptor" OR "age receptor" OR "advanced glycosylation end product-specific receptor" OR "ager protein" OR "RAGE" OR "sRAGE" OR "esRAGE" OR "FPS-ZM1" OR "PF-04494700" OR "corticoids" OR "Corticosteroids" OR "adrenal cortex hormones" OR "ketosteroids" OR "oxosteroids" OR "glucocorticoid" OR "glucorticoid effects" OR "glucocorticoids" OR "hydroxycorticosteroids" OR "hydroxycorticosteroid" OR "11-epicortisol" OR "50-23-7" OR "domolene" OR "cortisol" OR "cortifan" OR "wi4x0x7bpj" OR "barseb-hc" OR "epicortisol" OR "epicortisol" OR "komed-hc" OR "rectoid" OR "barseb hc" OR "(11beta)-11,17,21-trihydroxypregn-4-ene-3,20-dione" OR "heb-cort" OR "cortril" OR "hydrocortisone" OR "mineralocorticoids" OR "mineralocorticoid hormone" OR "mineralocorticoid effects" OR "1,2-dehydrocortisone" OR "encortone" OR "1,2-dehydrocortisone" OR "panasol" OR "deltacortisone" OR "decortisyl" OR "delta-cortisone" OR "decortin" OR "cortan" OR "rectodelt" OR "prednicen-m" OR "liquid pred" OR "vb0r961hzt" OR ".delta.1-cortisone" OR "predeltin" OR "delta 1-cortisone" OR "orasone" OR "prednidib" OR "prednisone" OR "panafcort" OR "dacortin" OR "cortancyl" OR "pred" OR "53-03-2" OR "deltasone" OR "pronisone" OR "encorton" OR "delta-dome" OR "dehydrocortisone" OR "prednisonum" OR "lisacort" OR "sterapred" OR "ultracorten" OR "winpred" OR "metacortandracin" OR "meticorten" OR "sone" OR "50-24-8" OR "predate" OR "prednisolone" OR "(11beta)-11,17,21-trihydroxypregna-1,4-diene-3,20-dione" OR "di adreson f" OR "deltahydrocortisone" OR "9phq9y1olm" OR ".delta.1-hydrocortisone" OR "prednisolonum" OR "sterane" OR "delta-f" OR "meti derm" OR "di-adreson-f" OR "metacortandralone" OR "delta(1)hydrocortisone" OR "predonine" OR "Inhaled sedation" OR "Halogenated agent" OR "Halogenated agents" OR "Halogenates" OR "Halogenate" OR "Volatile anesthetics" OR "Volatile agents" OR "Sevoflurane" OR "sevorane" OR "isoflurane" OR "Desflurane" OR anticoagulant* OR "fibrinolytic" OR "tissue factor pathway inhibitor" OR "lipoprotein-associated coagulation inhibitor" OR "Thrombin-thrombomodulin complex" OR "thrombin receptor" OR "activated protein C" OR "drotecogin alfa" OR "antithrombin" OR "plasminogen activator" OR "heparin")

**#3** (animal* OR experimental* OR "in vivo" OR "mice" OR "rats" OR "mouse" OR "pig" OR "pigs" OR piglet* OR "rabbit" OR "sheep*" OR swine* OR "dog" OR "dogs" OR "non-human primates" OR "ex vivo" )

**#4** ("therapy" OR "treatment*" OR therapeutic* OR "drug therapy" OR effect**:ti*OR "treat" OR "treating" OR "therapies" OR "treated" OR "treats" OR pharmacolog* OR "pharmacotherapy" )

**#1 AND #2 AND #3 AND #**
